# A Novel Method for Primary Blood Cell Culturing and Selection in *Drosophila melanogaster*

**DOI:** 10.3390/cells12010024

**Published:** 2022-12-21

**Authors:** Enikő Kúthy-Sutus, Bayan Kharrat, Erika Gábor, Gábor Csordás, Rita Sinka, Viktor Honti

**Affiliations:** 1 *Drosophila* Blood Cell Differentiation Group, Institute of Genetics, Biological Research Centre, P.O. Box 521, H-6701 Szeged, Hungary; 2 Faculty of Science and Informatics, Doctoral School of Biology, University of Szeged, P.O. Box 427, H-6720 Szeged, Hungary; 3 Lysosomal Degradation Research Group, Institute of Genetics, Biological Research Centre, P.O. Box 521, H-6701 Szeged, Hungary; 4 Department of Genetics, Faculty of Science and Informatics, University of Szeged, H-6726 Szeged, Hungary

**Keywords:** ex vivo culture, blood cells, *Drosophila melanogaster*, lamellocytes, transdifferentiation

## Abstract

The blood cells of the fruit fly *Drosophila melanogaster* show many similarities to their vertebrate counterparts, both in their functions and their differentiation. In the past decades, a wide palette of immunological and transgenic tools and methods have been developed to study hematopoiesis in the *Drosophila* larva. However, the in vivo observation of blood cells is technically restricted by the limited transparency of the body and the difficulty in keeping the organism alive during imaging. Here we describe an improved ex vivo culturing method that allows effective visualization and selection of live blood cells in primary cultures derived from *Drosophila* larvae. Our results show that cultured hemocytes accurately represent morphological and functional changes following immune challenges and in case of genetic alterations. Since cell culturing has hugely contributed to the understanding of the physiological properties of vertebrate blood cells, this method provides a versatile tool for studying *Drosophila* hemocyte differentiation and functions ex vivo.

## 1. Introduction

The regulation of hematopoiesis involves numerous signaling pathways, as well as epigenetic and transcription factors, which are highly conserved between the fruit fly *Drosophila melanogaster* and vertebrates. This high level of homology and the availability of a wide range of transgenic markers to trace blood cell lineages and genetic tools to easily manipulate the genome made the fruit fly *Drosophila melanogaster* an ideal organism for studying the differentiation of blood cells. The blood cells (hemocytes) of *Drosophila* are the functional counterparts of the myeloid immune cells of vertebrates [1,2,3,4,5,6,7]. Similarly, these cells are confined in hematopoietic compartments that evolve according to the developmental stage [3,8,9]. Such compartments in the larva are the lymph gland, a pair-lobed organ at the anterior part of the dorsal vessel, the sessile hematopoietic compartment consisting of hemocytes attached to the body wall, and the circulation [10,11,12,13,14,15].

The circulating blood cells of the *Drosophila* larva are categorized into three distinct classes: the phagocytic plasmatocytes, the melanizing crystal cells, and the large flat lamellocytes, which play an indispensable role in encapsulating invaders that are too large to be engulfed by plasmatocytes [4,16,17,18]. While plasmatocytes are predominant in the larval circulation, crystal cells are present in low numbers (~1–5%). Lamellocytes are rarely present in naive larvae but differentiate rapidly following immune induction, such as wasp parasitation or wounding [4,19,20,21]. These cells differentiate either from progenitors located in the lymph gland or via conversion from plasmatocytes, a process similar to the transdifferentiation of Th17 cells in vertebrates [14,21,22,23,24,25,26,27,28]. Dysregulation of signaling pathways controlling hematopoiesis can lead to lamellocyte differentiation without immune induction and, in some cases, cause the formation of melanotic nodules and a leukemia-like condition in the larva [11,29,30,31,32]. With these advantages in mind, it is not surprising that *Drosophila melanogaster* has emerged as a powerful model organism to study the genetic underpinnings and regulatory mechanisms of both healthy hematopoiesis and immune cell disorders.

To carry out such investigations, a wide variety of tools, including cell-type specific antibodies, transgenic reporters, as well as cutting-edge microscopy techniques, were developed [21,33,34,35,36,37,38,39,40]. While these approaches were instrumental to the description and characterization of the different blood cell classes, the observation of live hemocytes remains challenging. Hemocytes in the larva are often difficult to study through the cuticle, and larval movements are not easily controlled in experiments requiring longer observation periods. Although primary blood cell cultures would offer a solution to these challenges, attempts to establish such cultures have resulted in limited success. Larval blood cells were maintained ex vivo for brief periods of time to investigate lamellocyte transdifferentiation, but no long-term culturing conditions have been developed to date [24,40].

In this report, we describe an optimized method for the ex vivo culturing of *Drosophila* hemocytes. This method is suitable for studying blood cell function and differentiation, and it utilizes previously developed in vivo markers specific to the different hemocyte clusters. In these ex vivo cell cultures, hemocyte composition differs according to the genotype and the immune state of the larvae, and cells maintain their ability to proliferate and transdifferentiate. Furthermore, we developed a blasticidin resistance-based selection system similar to that previously utilized in vertebrate cell cultures. By employing the ex vivo culturing method, hemocytes originating from multiple reproducible conditions can be studied at the level of both morphology and transgenic marker expression. Moreover, the cell-type-specific selection allows the enrichment of specific hemocyte classes for downstream analysis.

## 2. Materials and Methods

### 2.1. Drosophila Stocks and Maintenance

All *Drosophila* lines used in the study are listed in Table S1. The flies were kept on a standard cornmeal-yeast media at 25 °C. All crosses were performed at 25 °C.

### 2.2. Wasp Infestation and Wounding

For wasp infestation, 25 *Drosophila* females were left to lay eggs for 6 h. 72 h after egg laying, early 3rd instar larvae were exposed to 25 female *Leptopilina boulardi* G486 parasitic wasps for 6 h at 25 °C. Hemocytes were isolated from infested larvae 16 h after infestation. Infested larvae were selected based on the melanized injury site caused by the oviposition.

Non-sterile mechanical wounding was performed on early 3rd instar larvae with a pin (Austerlitz Insect Pin 0.2 mm). Hemocytes were collected 16 h after immune induction for the ex vivo cell cultures.

### 2.3. Ex Vivo Culturing of Isolated Hemocytes

**Figure 1:**
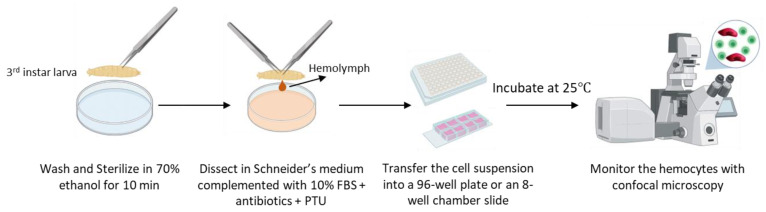
A schematic illustration of the *Drosophila* hemocyte culturing method.

To sterilize and remove food debris from the larval cuticle, 3rd instar larvae were washed out from the food into Millipore water, transferred into 70% ethanol for 10 min, and then to Millipore water again until dissection. To achieve optimal hemocyte confluency, 20 larvae were dissected from naive, wounded, and wasp-infested *Me* larvae, while 10 larvae were used from the tumorous genotypes (*Me,hop^Tum^*, *Me; l(3)mbn^1^* and *Me; Hml>Pvrλ*). The larvae were dissected in 200 μL of Schneider’s *Drosophila* medium (Lonza, Gampel, Switzerland, CatNo. 0000879623), complemented with 10% heat-inactivated Fetal Bovine Serum (Biosera, Nuaille, France, CatNo. FB-1090/500), 0.01 mg/mL gentamicin (BioConcept, Allschwil, Switzerland, CatNo. 4-07F00-H), 0.1 mg/mL Penicillin-Streptomycin (BioConcept, Allschwil, Switzerland CatNo. 4-01F00-H). To prevent hemocyte melanization, PTU (*N*-Phenylthiourea, Sigma-Aldrich, St. Louis, MO, USA, CatNo. P7629) was added to the medium. The medium containing the isolated hemocytes was carefully suspended with a sterile cut P200 tip (Greiner bio-one, Kremsmünster, Austria, CatNo. G739295), and 200 μL of the suspension was transferred into a well of a 96-well plate (Costar, Washington, DC, USA, CatNo. 3598). For the lamellocyte heterogeneity experiment, 15 larvae from naive, wounded, and wasp-infested conditions were dissected in 300 μL of culturing medium, and the cell suspension was transferred into a well of an 8-well chamber slide (Ibidi, Gräfelfing, Germany, CatNo. 80826). In all experiments, the cells were left to attach for 1 h before imaging at the Day 0 time point. Cell cultures were incubated at 25 °C, with the culturing medium being changed every two days. To visualize the nuclei, Hoechst 33342 (bisBenzimide H 33342 trihydrochloride, Sigma-Aldrich, St. Louis, MO, USA, CatNo. 14533) was added to the complemented Schneider’s medium at a 0.5 µg/mL final concentration. Images of cultured hemocytes were taken using Olympus Cell-R and Zeiss LSM800 microscopes (Figure 1).

### 2.4. Video Microscopy

Blood cells and wasp eggs were isolated from wasp-infested larvae 24 h after infestation. The cells were left to attach for 1 h, after which 48 h long videos were recorded using CytoSmart Lux3 FL (Axion BioSystems, Atlanta, GA, USA) fluorescence microscope.

### 2.5. Generation of the UAS-BsdR Line

The blasticidin resistance gene (*BsdR*) was amplified with PCR from pCoBlast vector using specific primers with 15-mer homology extension to the linearized vector end: 5′-ACTCTGAATAGGGAATTGGGATGGCCAAGCCTTTGTCTC-3′ and 5′-CGGCCGCAGATCTGTTAACGTTAGCCCTCCCACACATAAC-3′. The PCR fragment was cloned into the EcoRI (New England Biolabs, Ipswich, MA, USA, CatNo. R3101S) digested pUAST-attB vector with the SLIC cloning method [41]. The generated plasmid was injected into *Drosophila* embryos carrying a *vasa*-driven ΦC31 integrase and an attP40 site on the second chromosome. Afterward, a homozygous *Drosophila* line carrying the inducible blasticidin resistance transgene was established.

### 2.6. Blasticidin Treatment of Cell Cultures

Blood cells from 20 3rd instar larvae were isolated from each genotype, cultured in a 96-well plate, and allowed to adhere overnight. The next day, the culture medium was substituted with a selective medium containing 10 μg/mL blasticidin (Blasticidine S hydrochloride, Sigma-Aldrich, St. Louis, MO, USA, CatNo. 15205). Cells were cultured for 5 days, with the selective medium replaced every second day. For the *Hml>GFP* + *Hml-DsRed* co-culture experiment, blood cells from 10 *Hml-DsRed* larvae were mixed with blood cells from either 10 *Hml>GFP* larvae or 10 *Hml>GFP>BsdR* larvae, and the *Hml>GFP>BsdR* + *Hml-DsRed* culture was treated with blasticidin according to the protocol mentioned above.

### 2.7. Cell Counting, Cell Size Measurement and Statistics

Cell counting was performed manually using the multi-point tool in ImageJ/Fiji (US National Institutes of Health, Bethesda, MD, USA) image processing software. The nuclei were counted automatically using the ‘cellcounter’ macro in the same software. Measurement of lamellocyte size was performed using the ‘Measure Cell Surfaces’ macro in ImageJ/Fiji. Cells in three fields of view at a 10× objective were counted from each sample. Since different numbers of larvae were dissected from tumorous and non-tumorous genotypes, cell numbers are represented in percentages. For cell count in Figure S1, the number of cells was normalized for each condition by dividing the total cell number by the number of larvae and represented as fold change compared to the control mean. All experiments were repeated three times. Experiments were evaluated using Microsoft Excel and GraphPad Prism software version 6.0 for Windows. Graphs and illustrations were made using GraphPad Prism and BioRender online tools. Data were analyzed using analysis of variance (ANOVA) with Tukey’s test for multiple comparisons. Values of *p* ˂ 0.05 were accepted as significant (* *p* ≤ 0.05, ** *p* ≤ 0.01, *** *p* ≤ 0.001, **** *p* ≤ 0.0001). 

## 3. Results

### 3.1. Hemocyte Composition and Morphology in Primary Cell Cultures Depends on the Genotype and the Immune Condition of the Larva

To facilitate the long-term, high-resolution analysis of live *Drosophila* immune cells, we set out to establish primary blood cell cultures originating from larval hemocytes. We focused our attention on plasmatocytes and lamellocytes in order to study the predominant immune cell classes under homeostatic and immune-induced conditions. To visualize the different hemocyte types in the culture, we used the *Me* (*msnCherry,eaterGFP*) transgenic reporter combination, which marks lamellocytes with red and plasmatocytes with green fluorescence [21,38].

We analyzed the blood cell composition of hemocyte cultures derived from naive, wounded, and parasitized *Me* larvae on the day of isolation (Day 0) and six days later (Day 6). Furthermore, we assessed three previously described genetically induced leukemia models with hemocyte proliferation and lamellocyte differentiation phenotypes. The tested genotypes were *Me; l(3)mbn^1^*, in which the *l(3)mbn* tumor suppressor gene is mutant, *Me,hop^Tum^*, carrying constitutively active allele of *hopscotch* (*hop*), and *Me; Hml>Pvrλ,* where the constitutively active form of the receptor tyrosine kinase Pvr is expressed with the hemocyte specific *Hemolectin* (*Hml-Gal4*) driver [4,30,31,42]. All cultures could be maintained for six days regardless of the genotype and the immune condition, with cell number not differing significantly between Day 0 and Day 6 of culturing except for *Me; l(3)mbn^1^* and *Me,hop^Tum^* cultures (Figure S1).

**Figure 2:**
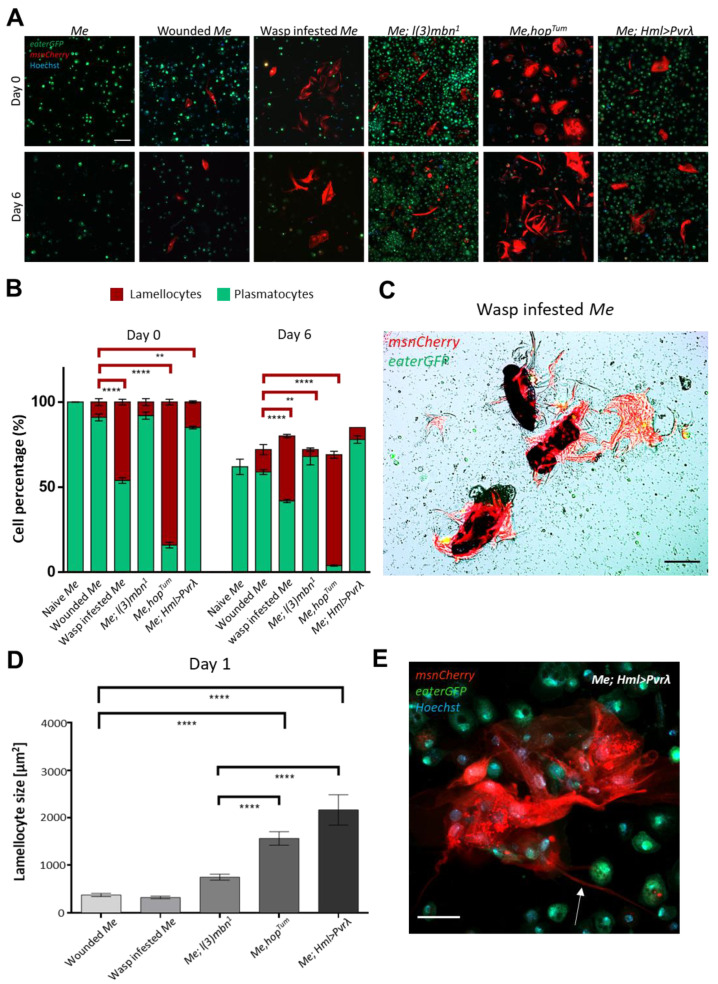
Hemocyte composition and morphology in primary larval blood cell cultures. (**A**) Representative images of hemocyte cultures of naive, wounded, and wasp-infested *Me*, and tumorous *Me; l(3)mbn^1^*, *Me,hop^Tum^* and *Me; Hml>Pvrλ* at the day of isolation (Day 0, 16 h after immune induction in case of wounded and wasp infested *Me*) and 6six days after (Day 6) (blue: nuclei, green: plasmatocytes, red: lamellocytes). Scale bar: 20 μm. (**B**) Bar graph showing the percentage of plasmatocytes and lamellocytes (ratio of GFP or Cherry positive cells on Day 0 or 6 to the GFP+Cherry positive cells on Day 0 in the same culture) in naive, wounded, and wasp infested *Me*, and tumorous *Me; l(3)mbn^1^*, *Me,hop^Tum^* and *Me; Hml>Pvrλ* cultures on Day 0 and Day 6. Cells in three fields of view at a 10× objective were counted from each sample (n = 3), and the data are the mean ± SD of three independent experiments. Data were analyzed using ANOVA with Tukey’s test for multiple comparisons, ** *p* ≤ 0.01, **** *p* ≤ 0.0001. (**C**) Lamellocytes on the surface of isolated parasitoid wasp eggs in a wasp-infested *Me* culture 48 h after culturing (green: plasmatocytes, red: lamellocytes). Scale bar: 100 μm. (**D**) Bar graph showing lamellocyte size in hemocyte cultures of wounded and wasp infested *Me*, and tumorous *Me; l(3)mbn^1^*, *Me,hop^Tum^* and *Me; Hml>Pvrλ*, data are mean ± SD of the size of 50 lamellocytes. Data were analyzed using ANOVA with Tukey’s test for multiple comparisons, **** *p* ≤ 0.0001. (**E**) Filopodium-like structures typical of lamellocytes in blood cell cultures of *Me; Hml>Pvrλ* larvae on Day 1 of culturing (blue: nuclei, green: plasmatocytes, red: lamellocytes, arrow indicates filopodium). Scale bar: 20 μm.

Investigation of the blood cell composition of these cultures revealed that, as expected, the control culture originating from naive 3rd instar larvae contained no lamellocytes either on Day 0 or Day 6 of culturing. However, in cultures derived from immune-induced and tumorous larvae, lamellocytes were observed at both time points (Figure 2A). Although a previous study by Márkus et al. (2005) [43] did not show a difference in lamellocyte differentiation depending on the mode of induction, in our experiments, immune induction with the parasitic wasp *Leptopilina boulardi* resulted in a significantly higher number of lamellocytes than wounding with an insect pin, with no difference in total cell number (Figure 2A,B and Figure S1). Moreover, in cultures derived from wasp-infested larvae, lamellocytes were present on the surface of the wasp egg two days after dissection in the case of both the wild type and the *hop^Tum^* mutant (Figure 2C, Supplementary Video S1), suggesting that lamellocytes can retain an attachment to the parasite ex vivo. Interestingly, similar to what has been reported previously in vivo [11,30,31], in cultures derived from tumorous larvae, total hemocyte number and lamellocyte percentage varied depending on the genotype. While wounded and wasp-infested *Me* cultures did not differ in cell number from the naive *Me* control, the three examined tumorous cultures (*l(3)mbn^1^*, *hop^Tum^* and *Hml>Pvrλ)* had a significantly higher cell number with *l(3)mbn^1^* and *hop^Tum^* cultures containing significantly more cells than *Hml>Pvrλ* (Figure S1), which resulted in a lower percentage of lamellocytes in *l(3)mbn^1^* (6%) than in *Hml>Pvrλ* cultures (~15%) (Figure 2A,B and Figure S1). *hop^Tum^* cultures, on the other hand, contained the highest percentage of lamellocytes (~90%) among the investigated conditions, in addition to a very high total cell number, highlighting the severity of the immune phenotype in these mutants (Figure 2A,B and Figure S1).

Moreover, we also compared the size of lamellocytes in the different cultures after one day. While we found no significant difference in lamellocyte size between cultures originating from wounded, wasp parasitized and *l(3)mbn^1^* larvae, lamellocytes derived from *hop^Tum^* and *Hml>Pvrλ* tumourous larvae were significantly larger in size (Figure 2D). Interestingly, lamellocytes of *Hml>Pvrλ* larvae were not only bigger but also exhibited long filopodia or cytoneme-like structures (Figure 2E). This all indicates that continuous activation of signaling pathways, as in the case of *hop^Tum^* and *Hml>Pvrλ* tumorous genotypes, affects not only the number but also the size and morphology of the differentiated lamellocytes.

Taken together, the above demonstrates that immune cell number and composition in ex vivo cultures correlate to the larval immune state and reflect changes induced by immune stimuli or misregulation of regulatory signaling pathways. Our observations also indicate that hemocytes ex vivo mostly retain their number and cell type composition even after six days of culture. We thus propose that the ex vivo conditions can be used in future experiments to monitor hemocyte number, characteristics, and morphology for up to one week after a certain genetic manipulation or drug treatment.

### 3.2. Cultured Hemocytes Maintain Their Proliferation and Transdifferentiation Capacity

The ability of plasmatocytes to undergo cell division is one of the hallmarks of this cell type [8,13]. To investigate whether cultured hemocytes proliferate, we isolated blood cells from naive, wounded, and wasp-parasitized *Cg*>*Fly-FUCCI* larvae, in which the in vivo cell cycle indicator is expressed by a pan-hemocyte driver *Collagen* (*Cg-Gal4*) [44,45]. In all three cultures, hemocytes were able to undergo cell division even on the fourth day of the experiment. Unexpectedly, we did not observe a significant difference in cell cycle among the tested immune conditions. This is possibly due to the fact that cultured hemocytes lose the immune trigger present in wounded and parasitized larvae and thus revert to their default developmental proliferation rate.

**Figure 3:**
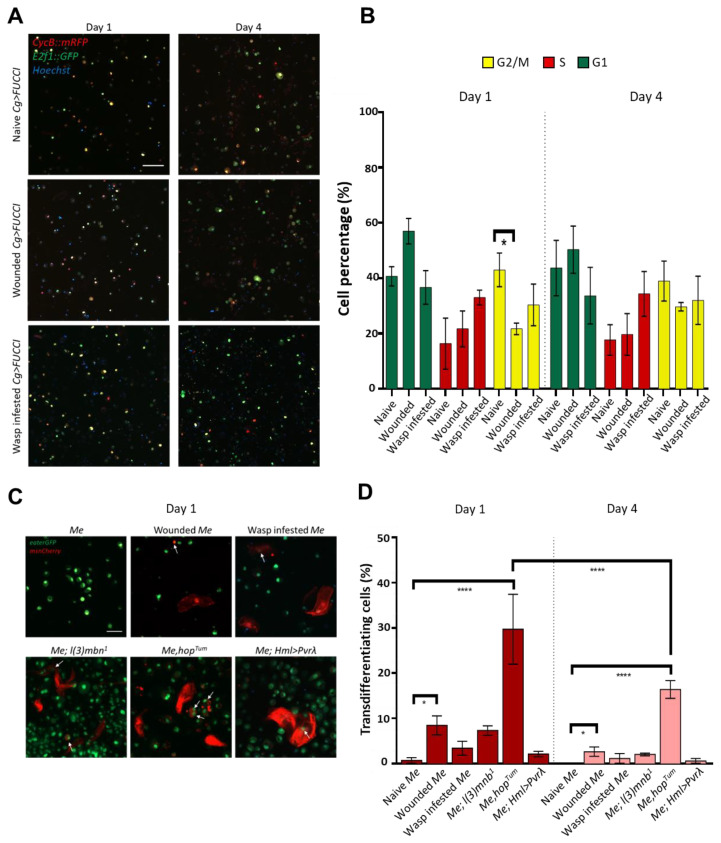
Proliferation and transdifferentiation in ex vivo hemocyte cultures. (**A**) Representative images of naive, wounded, and wasp-infested *Cg*>*Fly-FUCCI* cultures on Day 1 and Day 4 (green: cells in G1 phase, red: cells in S phase, yellow: cells in G2/M phase). Scale bar: 50 μm. (**B**) Bar graph showing the percentage of cells in G1, S, and G2/M phase in hemocyte cultures derived from naive, wounded, and wasp-infested *Cg*>*Fly-FUCCI* larvae. Cells in three fields of view at a 10× objective were counted from each sample (n = 3), and the data are the mean ± SD of three independent experiments. Data were analyzed using ANOVA with Tukey’s test for multiple comparisons, * *p* ≤ 0.05. (**C**) Representative images of hemocyte cultures of naive, wounded and wasp infested *Me*, and tumorous *Me; l(3)mbn^1^*, *Me,hop^Tum^* and *Me; Hml>Pvrλ* on Day 1 of culturing (green: plasmatocytes, red: lamellocytes), arrows indicate transdifferentiating cells expressing both *eaterGFP* and *msnCherry*. Scale bar: 20 μm. (**D**) Bar graph showing the percentage of transdifferentiating cells (ratio of *eaterGFP* and *msnCherry* double positive cells to the total cell number in the culture) in naive, wounded, and wasp infested *Me*, and tumorous *Me; l(3)mbn^1^*, *Me,hop^Tum^* and *Me; Hml>Pvrλ* on Day 1 and Day 4 of culturing. Cells in three fields of view at a 10x objective were counted from each sample (n = 3), and the data are the mean ± SD of three independent experiments. Data were analyzed using ANOVA with Tukey’s test for multiple comparisons, * *p* ≤ 0.05, **** *p* ≤ 0.0001.

Following immune challenges and under tumorous conditions, a fraction of circulating plasmatocytes convert into lamellocytes in a process called transdifferentiation [4,21,23,24,27,28]. In these cultures, we indeed observed hemocytes showing intermediate morphology between plasmatocytes and lamellocytes (Figure 3C). These cells expressed the plasmatocyte-specific *eaterGFP* and the lamellocyte-specific *msnCherry* markers simultaneously, which suggests they are either “double-positive” type II lamellocytes (as described by Anderl et al., 2016 [21]) originating from *eaterGFP* expressing cells or plasmatocytes undergoing conversion into lamellocytes. As both of these types originate from *eaterGFP* hemocytes, we considered them transdifferentiating cells. We found that the ratio of these cells to the total cell number on Day 1 was the highest in the *Me,hop^Tum^* culture (~29%), followed by cultures derived from wounded *Me* (~8%), then *Me; l(3)mbn^1^* (~7%), with wasp-infested *Me* and *Me; Hml>Pvrλ* containing the lowest ratio of transdifferentiating cells, (~3%) and (~2%), respectively (Figure 3C,D). Interestingly, even on Day 4, transdifferentiating cells could be found in all cases except for naive *Me* controls, with a significant reduction in their ratio in *Me,hop^Tum^* cultures. These findings align with the previous results that *hop^Tum^* cultures are characterized by an extremely high lamellocyte ratio, which increases further on Day 6 (Figure 2A,B), and highlight more how the mode of induction or the genetic background affects the lamellocyte differentiation process.

Taken together, our results suggest that hemocytes preserve their ability to proliferate and transdifferentiate in hemocyte cultures, which makes ex vivo cultures suitable for studying factors playing a role in these processes.

### 3.3. Primary Hemocyte Cultures Contain Heterogeneous Lamellocyte Subsets

**Figure 4:**
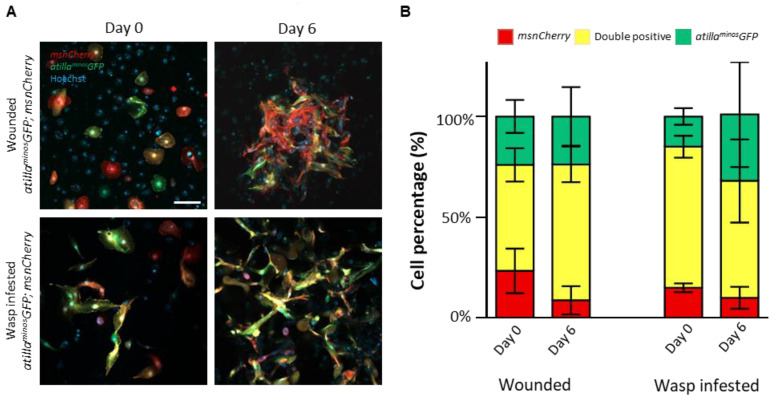
Lamellocyte composition in wounded and wasp-infested *atilla^minos^GFP*; *msnCherry* hemocyte cultures. (**A**) Representative images of lamellocytes in wounded and wasp-infested *atilla^minos^GFP*; *msnCherry* on Day 0 and Day 6 of culturing (green: *atilla^minos^GFP*, red: *msnCherry*, blue: nuclei). Scale bar: 100 μm. (**B**) Bar graph showing the percentage of *Cherry* positive, GFP positive, and GFP+Cherry double positive lamellocytes in wounded and wasp infested *atilla^minos^GFP*; *msnCherry* cultures on Day 0 and Day 6 of culturing. Cells in three fields of view at a 10x objective were counted from each sample (n = 3), and the data are the mean ± SD of three independent experiments. Data were analyzed using ANOVA with Tukey’s test for multiple comparisons.

As we observed variation in lamellocyte morphology among the tested conditions and the presence of a subset of lamellocytes that express *eaterGFP* plasmatocyte marker, we were intrigued to know whether the observed lamellocytes represented heterogeneous cell populations or various states of lamellocyte differentiation. To investigate this, we combined the two most commonly used lamellocyte markers, Atilla/L1 (*atilla^minos^GFP* [37]) and Misshapen (*msnCherry* [38]), in a single fly line (*atilla^minos^GFP*; *msnCherry*), and isolated blood cells from 3rd instar larvae after wounding and wasp infestation. By observing the hemocytes on Day 0 and Day 6 of culturing, we found that in both cultures at both time points, lamellocytes expressing all possible combinations of reporters (GFP only, Cherry only, and GFP-Cherry double positive) were present. However, the relative abundance of these lamellocyte populations did not depend on the mode of induction or the day of observation (Figure 4A,B). These results suggest that *atilla^minos^GFP* and *msnCherry* expression may not represent specific maturation states of lamellocytes but rather mark two overlapping lamellocyte subsets that persist over time.

### 3.4. Construction of a Blasticidin Resistance-Based Blood Cell Selection System

**Figure 5:**
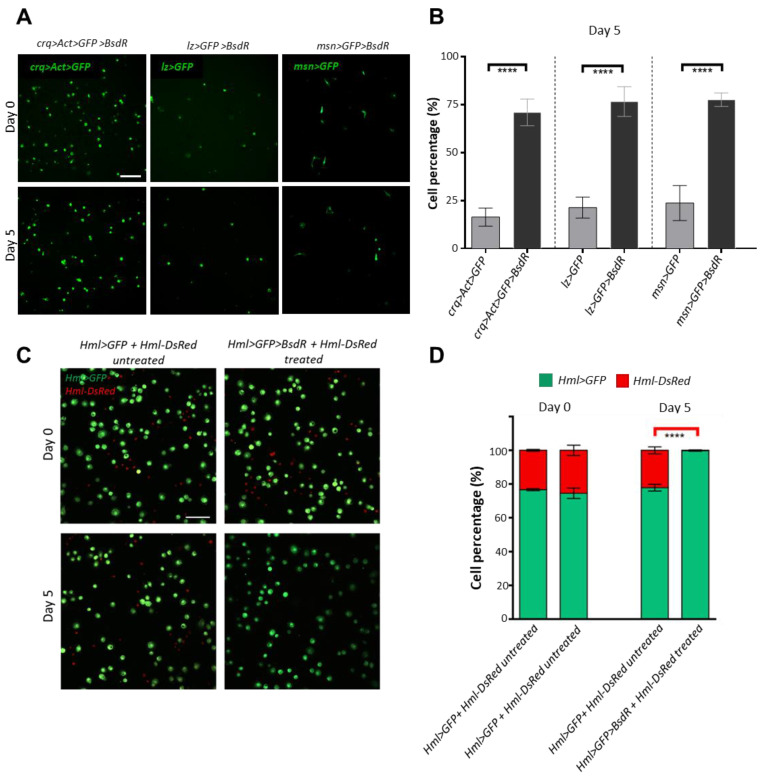
A blasticidin selection system for hemocyte subsets cultured ex vivo. (**A**) Representative images of *crq>Act>GFP>BsdR*, *lz>GFP>BsdR* and *msn>GFP>BsdR* cultures on Day 0 and Day 5 after treatment with blasticidin (green: *crq>Act>GFP*, *lz>GFP* and *msn>GFP,* respectively). Scale bar: 100 μm. (**B**) Bar graph showing the percentage of surviving cells (ratio of GFP positive cells on Day 5 to the GFP positive cells on Day 0 in the same culture) after blasticidin treatment in *crq>Act>GFP*, *crq>Act>GFP>BsdR, lz>GFP*, *lz>GFP>BsdR*, *msn>GFP*, and *msn>GFP>BsdR* cultures. Cells in three fields of view at a 10× objective were counted from each sample (n = 3), and the data are the mean ± SD of three independent experiments. Data were analyzed using ANOVA with Tukey’s test for multiple comparisons, **** *p* ≤ 0.0001. (**C**) Representative images of untreated *Hml>GFP* + *Hml-DsRed* and treated *Hml>GFP>BsdR* + *Hml-DsRed* cultures on Day 0 and Day 5 of culturing. (green: *Hml>GFP*, red: *Hml-DsRed*). Scale bar: 50 μm. (**D**) Bar graph showing the percentage of surviving *Hml>GFP* and *Hml-DsRed* cells (ratio of GFP or DsRed positive cells, respectively, to the total GFP+DsRed positive cells in the same culture) in untreated *Hml>GFP* + *Hml-DsRed* and treated *Hml>GFP>BsdR* + *Hml-DsRed* cultures on Day 0 and Day 5 of culturing. Cells in three fields of view of a 10× objective were counted from each sample (n = 3), and the data are the mean ± SD of three independent experiments. Data were analyzed using ANOVA with Tukey’s test for multiple comparisons, **** *p* ≤ 0.0001.

In addition to distinct transgenic markers that can be used to follow the different hemocyte subsets, *Drosophila* blood cell classes can also be distinguished by the expression of cell type-specific *Gal4* drivers [46]. Using these drivers in ex vivo cultures to drive UAS-regulated resistance factors would provide an opportunity to select and enrich specific hemocyte subsets for further investigations. Therefore, we generated *Drosophila* lines carrying a *UAS-Blasticidin Resistance* (*UAS-BsdR*) transgene. We used this resistance transgene in combination with various drivers that represent either a subset of plasmatocytes that originate from circulating/sessile hemocytes (*crq>Act>Gal4*) [23], crystal cells (*lz-Gal4*) or lamellocytes and lamellocyte precursors (*msn-Gal4*). Blood cells were isolated from naive larvae, except for the *msn-Gal4* driver, where, in order to induce lamellocyte differentiation, we wounded the larvae and collected the hemocytes 16 h after wounding. We treated the cells with blasticidin on the second day of culturing and cultured them for up to five days in a blasticidin-selective medium. When comparing the number of cells expressing GFP alone or GFP with *BsdR* on Day 5, we found that most of the cells expressing the *BsdR* survived (*crq>Act>GFP>BsdR:* ~71%, *lz>GFP>BsdR*: ~77%, *msn>GFP>BsdR*: ~77%), unlike cells that did not express *BsdR* in control cultures (*crq>Act>GFP*: ~16%, *lz>GFP*: ~21%, *msn>GFP*: ~23%) (Figure 5A,B and Figure S2). This suggests that the *UAS-BsdR* transgene can be used with *Gal4* drivers to select a specific cell type in cultures.

To further validate the ex vivo blasticidin selection system, we co-cultured BsdR-expressing *Hml>GFP* hemocytes with sensitive *Hml-DsRed* cells [4,15] and, from the second day of culturing, kept them on a blasticidin selective medium for five days. As expected, we found that *Hml-DsRed* cells were abolished completely from the treated culture on Day 5, and the cultures almost exclusively consisted of *Hml>GFP>BsdR* cells (Figure 5C,D). Together, these results show that hemocyte subpopulations can be successfully maintained and selected ex vivo using the blasticidin resistance system. This approach would allow further downstream investigations, such as bulk mRNA sequencing or proteomic studies on specifically enriched hemocyte subpopulations, without the need for FACS-sorting or single-cell sequencing.

## 4. Discussion

Cell cultures have served as a powerful tool for studying various biological events for decades [47,48,49,50,51,52,53]. The main advantage of using either primary or immortalized cell lines is that they provide a simplified environment to model biological conditions. Therefore, several cell lines have been established from common model organisms, and many of these are also commercially available [54,55,56,57,58,59,60,61,62,63,64,65].

Researchers using *Drosophila melanogaster* as a model organism can choose from more than 150 unique cell lines depending on the focus of their research [66,67,68,69,70,71,72,73,74]. Embryonic mesodermal cell lines that mostly resemble hemocytes, such as Kc 167, S2, or l(2)mbn, express innate immune response genes and are thus ideal for investigating host-pathogen interactions and identifying new drug targets [67,75,76]. Still, they are not suitable for studying the differentiation and gene expression dynamics of larval hemocytes.

Here, we describe an improved ex vivo culturing approach to maintain and investigate *Drosophila* larval immune cells. Unlike previous attempts [24,40], our experimental system is suitable for maintaining hemocytes for up to a week. We found that ex vivo cultured hemocyte number and cell types accurately represent the immune state of larvae at the time of isolation. In line with this, hemocytes isolated from naive larvae maintained their uninduced state, suggesting that the isolation procedure itself does not trigger an immune response that would affect our observations in vitro. It is worth noting that total cell numbers decreased over time, which could suggest that the described conditions can be further optimized.

Benefiting from the ex vivo cultures, we observed that infestation by the parasitic wasp *L. boulardi* results in a higher number of lamellocytes, more dividing cells, and fewer transdifferentiating cells than wounding with an insect pin, with no significant effect on total cell number or lamellocyte size. Recently, Evans and colleagues described that epidermal wounding activates Toll signaling in hemocytes via the production of ROS [76], leading to lamellocyte differentiation. In the case of parasitic wasp infection, the secretion of immunosuppressive venom and the presence of the parasitic eggs inside the larvae, which represent hemocyte attachment surfaces allowing local transdifferentiation, may compound this initial trigger, leading to a difference in immune response [21,77,78].

Furthermore, the maintenance of hemocytes originating from tumorous larvae also revealed that, in line with Zettervall et al., 2004 [31], both total cell numbers and the relative abundance of plasmatocytes and lamellocytes depend on the genotype causing the hematopoietic malignancy. For example, cultures from *hop^Tum^* larvae contained very few plasmatocytes throughout their culturing, while they were highly abundant in lamellocytes. In contrast, the *l(3)mbn^1^* mutation resulted in a similarly high overall cell number, but the cultures predominantly contained plasmatocytes. The fact that lamellocytes could be found even after six days of culture, irrespective of what triggered their differentiation, is intriguing. Since adult animals developing from parasitized larvae do not harbor lamellocytes in their circulation, they were assumed to be a relatively transient, albeit terminally differentiated cell type [8,79]. Moreover, the lamellocytes that were induced by the *hop^Tum^* mutation or the *Hml>Pvrλ* transgene combination were bigger in size and showed more cellular protrusions compared to their counterparts induced by wounding parasite-infestation or the *l(3)mbn^1^* mutation. This difference may be due to the independent effects the genetic background exerts on the cytoskeleton or the signaling pathways regulating cellular morphology.

Culturing immune cells also gave us the opportunity to investigate if the ex vivo environment is suitable for studying hemocyte proliferation and differentiation. We found that isolated hemocytes retained their ability to proliferate and transdifferentiate after four days of culturing. These results suggest that the observed immune cell proliferation and differentiation may be due to an intrinsic program that represents the immune state of the larvae when the hemocytes were isolated.

We used the ex vivo hemocyte cultures to study the composition of the lamellocyte subsets and to investigate whether the expression of the two previously identified *atilla* and *misshapen* lamellocyte markers change during lamellocyte maturation. However, our results showed no significant differences in the expression of these markers between Day 0 and Day 6 in cultures derived from both wounded and parasitized larvae. Although previous reports stated that during lamellocyte maturation, the level of *atilla* expression increases [28], we could not observe this in culture. This may be due to the higher sensitivity of single-cell sequencing to changes in gene expression than transgenic fluorescent reporters. Further investigation of the recently identified lamellocyte markers [27,28] under these culturing conditions would be essential for the characterization of the newly described lamellocyte subsets and for identifying factors that trigger their differentiation.

Finally, to facilitate cell-type-specific downstream analyses, we generated a *Drosophila* line with a UAS-driven blasticidin resistance transgene. We adapted this technique since using antibiotic-resistance genes to select dedicated cell subsets is a popular approach in mammalian cell culture investigations. The blasticidin selection system, in particular, was shown to be effective in a wide range of mammalian cell lines [80,81,82,83,84]. We observed that hemocytes expressing the resistance gene reliably survived after blasticidin treatment, while cells lacking the resistance gene expression did not. During the past years, several Gal4 drivers were generated that are expressed by specific hemocyte subsets. In addition, recent single-cell transcriptomic studies highlighted several potential genes whose expression is characteristic of hemocyte subclusters [7,27,28,85,86] and thus can also be utilized to create new driver lines. It is, therefore, tempting to speculate if these subpopulations could be maintained and enriched for further analyses ex vivo via specific expression of the blasticidin resistance transgene.

In vitro culturing and differentiation of immune cells provided us with fundamental knowledge of mammalian hematopoiesis, as well as valuable data on blood cell lineages, hematopoietic regulatory mechanisms, and therapeutic approaches. Based on the results presented in this report, we suggest that adapting a similar approach in fly immune cells, combined with the extensive genetic toolkit available in *Drosophila*, could significantly improve our understanding of hemocyte differentiation. Recent advances in conditional gene expression, optogenetics, and improved lineage tracing techniques, together with the blasticidin selection system, would allow the hand-picking of a single hemocyte subtype or, potentially, a single hemocyte. Within the selected population, gene expression or RNA interference could be induced while monitoring reporter expression changes with high-resolution microscopy. These experiments could ultimately point us toward new genes of interest or drug candidates, which could be investigated in healthy or pathologic hematopoiesis in humans.

## 5. Conclusions

In this report, we have developed a new culturing system that enables researchers to monitor blood cell development and function ex vivo and to selectively maintain hemocyte subsets with inducible blasticidin resistance, similar to the strategy employed in mammalian cell cultures. By examining cultures derived from larvae with distinct genotypes and immune conditions, we found that these cultures mirror the physiological changes that occur in the larva upon immune activation and that cultured hemocytes retain their ability to divide and transdifferentiate for up to a week. With the described culturing conditions, researchers can investigate more closely how blood cell development and transdifferentiation are regulated and can screen for modulators of these processes, which might be of interest for therapy against blood cell malignancies.

## Data Availability

Not applicable.

## Supplementary Materials

The following supporting information can be downloaded at: https://www.mdpi.com/article/10.3390/cells12010024/s1, Figure S1: Bar graph showing fold change of total cell number in cultures of naive, wounded and wasp infested *Me*, and tumorous *Me; l(3)mbn1*, *Me,hopTum* and *Me;* Hml>*Pvrλ* on Day 0 and Day 6 of culturing; Figure S2: Blasticidin treatment of sensitive hemocytes; Table S1: List of *Drosophila* lines [87,88]; Video S1: Time-lapse movie of lamellocytes on the surface of the wasp eggs. (**A**) *Me* lamellocytes on the surface of the wasp eggs 24 h after infestation, scale bar: 200 μm. (**B**) Lamellocytes originating from the wasp-infested *Me,hop^Tum^* on the surface of the wasp eggs 24 h after infestation. Scale bar: 200 μm.
