# Supplementary material for: A Novel Method for Primary Blood Cell Culturing and Selection in Drosophila melanogaster
Source: Cells. 2022 Dec 21;12(1):24. doi: 10.3390/cells12010024 (PMC9818912; doi:10.3390/cells12010024)

Table S1

Abbreviation	Genotype	Source
Me	*w,msnF9MO-Cherry,eater-GFP*	Reporters generated in Tokusumi et al., 2009 [38], combined in Anderl et al., 2016 [21]
Me; l(3)mbn^{1}/TM6,Tb	*w,msnF9MO-Cherry,eater-GFP; l(3)mbn^{1}/TM6,Tb*	Generated from combining *Me* with *l(3)mbn^{1} /TM6,Tb* (Konrad et al., 1994 [42])
Me,hop^{Tum}	*w,msnF9MO-Cherry,eater-GFP,hop^{Tum-l}*	Generated from combining *Me* with a homozygous viable *hop^{Tum}* strain (BDSC#8492)
Me; UAS-Pvrλ	*w,msnF9MO-Cherry,eater-GFP; UAS-Pvrλ*	Generated from combining *Me* with *UAS-Pvrλ* (Duchek et al., 2001 [87])
Hml-Gal4 (R3-Hml-Gal4)	*w; Hml-Gal4.Δ,UAS-2XEGFP*	Honti et al., 2014 [4]
crq>Act>Gal4	*y,w,UAS-FLP; crq-Gal4, Act5C-FRT-y^{+}-FRT GAL4,UAS-GFP*	Honti et al., 2010 [23]
lz-Gal4	*y,w,lz-Gal4,UAS-GFP*	A gift from Bruno Lemaitre, EPFL Lausanne
msnCherry; atilla^{minos}GFP	*w,msnF9MO-Cherry; Mi{ET1}atilla^{MB03539}*	Generated from combining *msnCherry* and *atilla^{minos}GFP* (Honti et al., 2009 [37])
msn>GFP	*w, UAS-mCD8::GFP; msn-Gal4*	Generated from crossing *msn-GAL4* (Lam et al., 2010 [88], a gift from Professor Dan Hultmark, Umea University) to *UAS-mCD8GFP* (a gift from József Mihály, BRC Szeged)
Cg>FUCCI	*w; Cg-Gal4; UAS-EGFP::E2F1$^{1-230}$,UAS-mRFP1::CycB$^{1-266}$*	Generated from combining *Cg-Gal4* (BDSC#7011) with *UAS-FlyFUCCI* (Zielke et al., 2014 [44], BDSC#55122)
Hml-DsRed	*W; HmlΔDsRed.nls*	Makhijani et al., 2011 [15]
UAS-BsdR	*w; UAS-BsdR{attP40}*	Generated in this study
vas-int; attP40	*y w M(eGFP, vas-int, dmRFP)ZH-2A; P{CaryP}attP40*	Fly Facility, Department of Genetics, University of Cambridge (https://www.ncbi.nlm.nih.gov/pmc/articles/PMC1805588/)

Figure S1

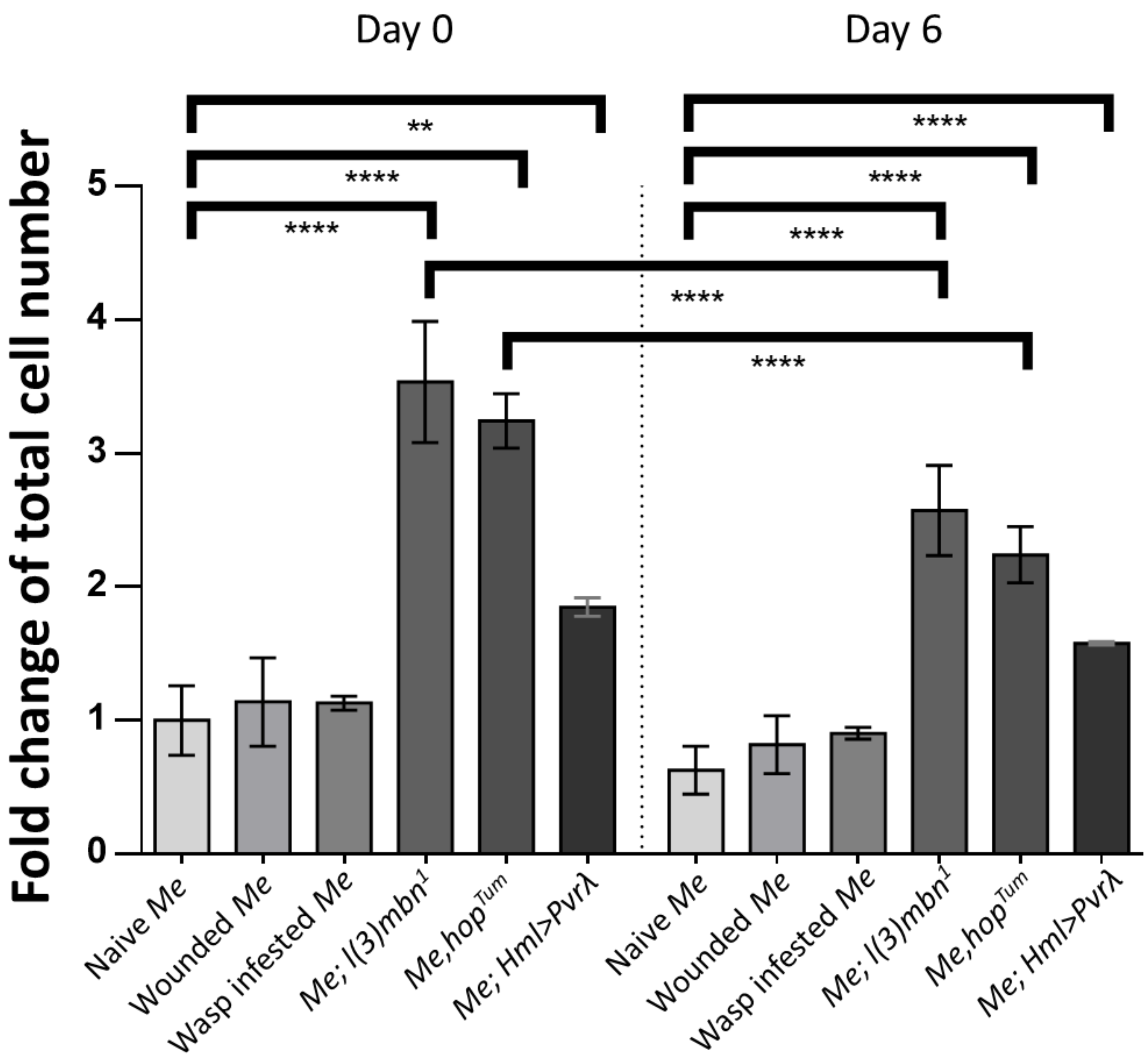

Day 0
Day 6
**
****
****
****
****
****
****
****
****
Fold change of total cell number
0
1
2
3
4
5
Naive Me
Wounded Me
Wasp infested Me
Me; l(3)mbn1
Me,hopTum
Me; Hml>Pvrλ
Naive Me
Wounded Me
Wasp infested Me
Me; l(3)mbn1
Me,hopTum
Me; Hml>Pvrλ

Figure S2

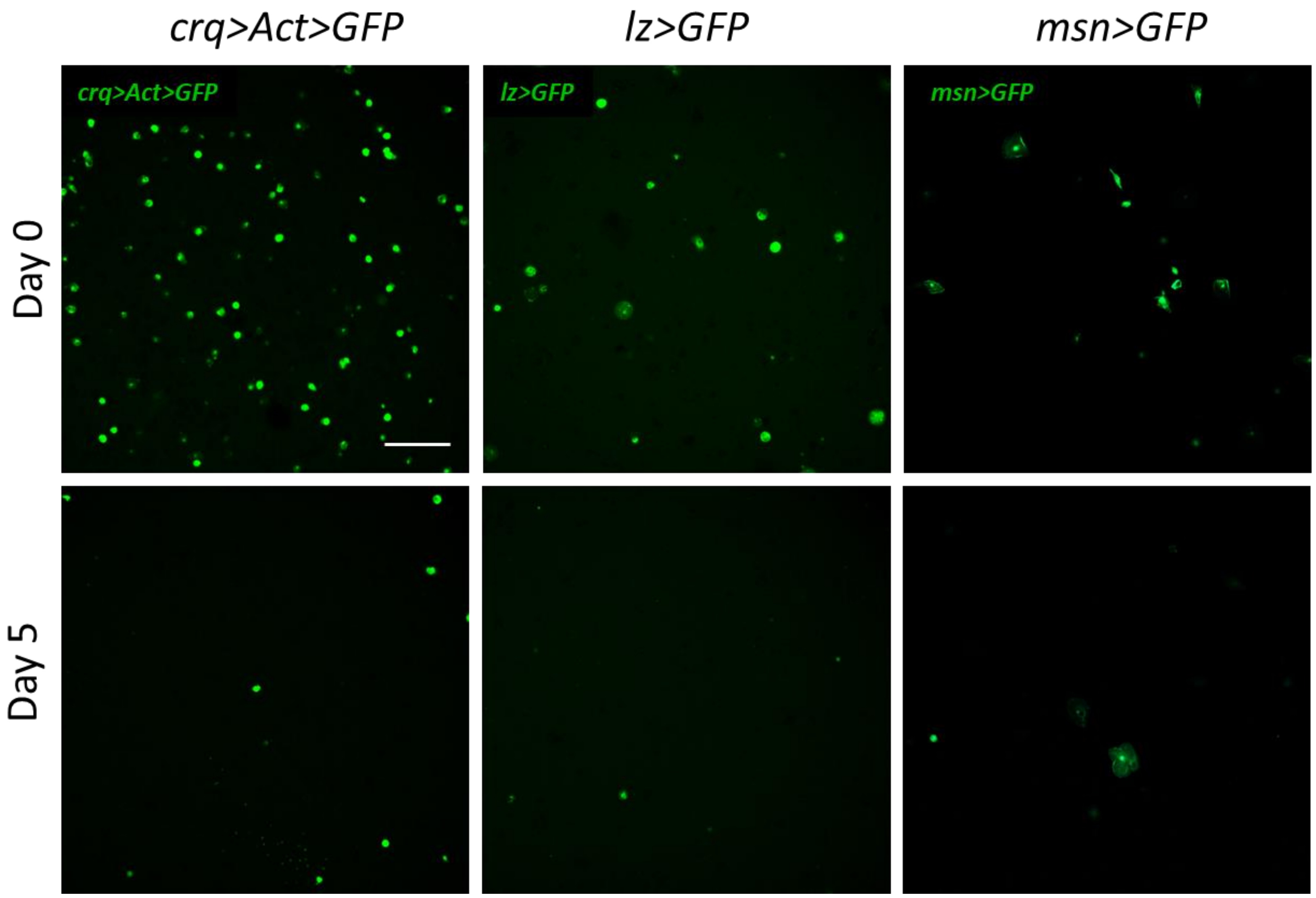

Supplement: Supplementary file 1 [file cells-12-00024-s001.zip › sup_figures 1 and 2 Sutus paper proof.pdf]
